# Capecitabine plus oxaliplatin as first-line treatment in patients with advanced biliary system adenocarcinoma: a prospective multicentre phase II trial

**DOI:** 10.1038/sj.bjc.6604178

**Published:** 2008-01-08

**Authors:** O Nehls, H Oettle, J T Hartmann, R-D Hofheinz, H G Hass, M S Horger, U Koppenhöfer, A Hochhaus, J Stieler, J Trojan, M Gregor, B Klump

**Affiliations:** 1 Department of Internal Medicine I, University Hospital, Tübingen, Germany; 2 Department of Hematology and Oncology, Charité, Campus-Virchow-Clinic, Berlin, Germany; 3 Department of Internal Medicine II and Center of Gastrointestinal Oncology (ZGO), University Hospital, Tübingen, Germany; 4 III. Medizinische Klinik, Medizinische Fakultät Mannheim der Universität Heidelberg, Mannheim, Germany; 5 Department of Diagnostic Radiology, University Hospital, Tübingen, Germany; 6 Department of Internal Medicine II, University Hospital, Frankfurt, Germany

**Keywords:** capecitabine, chemotherapy, cholangiocarcinoma, gallbladder carcinoma, oxaliplatin

## Abstract

This prospective multicentre phase II study characterises the toxicity and activity of first-line capecitabine and oxaliplatin combination therapy (CAPOX) in advanced biliary system adenocarcinomas. Patients received oxaliplatin (130 mg m^−2^, day 1) plus capecitabine (1000 mg m^−2^ b.i.d., days 1–14) every 3 weeks. Patients were stratified prospectively into two groups based on location of the primary (gallbladder carcinoma (GBC) or extrahepatic cholangiocarcinoma (ECC) *versus* intrahepatic mass-forming type cholangiocarcinoma (ICC)). Sixty-five patients were evaluable. The response rate in 47 patients with GBC/ECC was 27% (4% complete responses), and in 23 patients (49%) stable disease (SD) was encountered. In 18 patients with ICC, we observed no objective responses, but 6 patients (33%) had SD. Median survival was 12.8 months (95% CI, 10.0–15.6) for patients with GBC or ECC (GBC: 8.2 months; 95% CI, 4.3–11.7; ECC: 16.8 months; 95% CI, 12.7–20.5), and 5.2 months (95% CI, 0.6–9.8) for ICC patients. In both cohorts, therapy was well tolerated. The most common grade 3–4 toxicity was peripheral sensory neuropathy (11 patients). Our data suggest that the CAPOX regimen is a well-tolerated and active treatment option for advanced ECC and GBC but might produce poorer results for ICC.

Biliary system adenocarcinomas include clearly defined gallbladder carcinomas (GBC) as well as two main types of cholangiocarcinomas (CCCs): an infiltrative ductal type involving extrahepatic (ECC) and in some cases intrahepatic bile ducts, and an intrahepatic mass-forming type (ICC). These morphologically distinct presentations have been shown not only to differ at the molecular level, but also appear to be distinct in clinical behaviour and with regard to response to cytotoxic drugs (Jarnagin *et al*, 2006).

To date, complete surgical resection provides the only curative treatment option in the early stages of these neoplasms irrespective of their origin within the biliary system. However, less than one-third of CCCs are resectable, and patients presenting with UICC stage IV GBC or with unresectable CCC treated only by best supportive care have a poor prognosis with a median survival time of less than 6 months (Parker *et al*, 1996; Pitt *et al*, 1997; Jarnagin *et al*, 2001; Weber *et al*, 2002).

At present, there is no established palliative standard of care for GBC and CCC, and the cytotoxic agents most extensively studied, as single agents are 5-fluorouracil (5-FU), gemcitabine, capecitabine, mitomycin C, cisplatin, doxorubicin, and methotrexate, resulting in partial responses (PRs) in about 10–20% of selected patients (Hejna *et al*, 1998; Penz *et al*, 2001). However, some phase-II trials indicate that the use of combination therapies with newer agents may boost response rates to 26% and higher (Knox *et al*, 2005; Harder *et al*, 2006).

To date, the only randomised trial for advanced biliary carcinomas, including an observational control arm, has shown an improvement of overall survival (OS) (6.5 *versus* 2.5 months) as well as quality of life, assessing a regimen of 5-FU plus leucovorin with or without etoposid, as compared to best supportive care (Glimelius *et al*, 1996).

In this setting, other treatment modalities, such as photodynamic therapy, have yielded preliminary beneficial results only for patients with ECC due to locoregional tumour control but not for those with metastatic disease (Ortner *et al*, 1998). A survival benefit of photodynamic therapy over best supportive care has only been demonstrated for patients not amenable to conventional stenting procedures (Ortner *et al*, 2003); therefore, its value relative to conventional stenting procedures remains unclear. Recently performed targeted therapy approaches with the Her/2-neu and/or EGFR inhibitors lapatinib or erlotinib have shown no or only modest activity (0 and 8% responses, respectively) in advanced biliary system adenocarcinomas (Philip *et al*, 2006; Ramanathan *et al*, 2006).

Thus, a rational basis for the treatment of advanced stages of biliary system adenocarcinomas might only be provided by cytotoxic treatment alone or in the context of a multimodality therapy strategy, including chemotherapy.

Oxaliplatin (l-OHP), an alkylating diaminocyclohexane platinum derivate, has been noted to display a marked cytotoxic synergism in combination with fluoropyrimidines against a variety of solid human tumour cells (Raymond *et al*, 1997).

Therefore, we recently have conducted a prospective phase II study of two-weekly oxaliplatin plus high-dose 5-FU/folinic acid in biliary system adenocarcinomas. The disease control rate (responses and stable disease (SD)) was 56%, and the median OS was 9.5 months (Nehls *et al*, 2001). To improve efficacy and to offer a more convenient treatment option for patients by reducing clinical visits and avoiding indwelling devices, we prospectively investigated the activity and toxicity profile of three-weekly intravenous oxaliplatin plus oral capecitabine (CAPOX) in two stratified cohorts with either advanced GBC and ECC (group A) or advanced ICC (group B).

## PATIENTS AND METHODS

### Eligibility criteria

This prospective, four-institutional study, which was officially accredited by the Arbeitsgemeinschaft Internistische Onkologie (AIO), enrolled 66 consecutive patients with the diagnosis of biliary system adenocarcinoma between February 2002 and January 2004.

Criteria for inclusion were histologically or cytologically confirmed adenocarcinomas of the gallbladder or the intrahepatic or extrahepatic biliary tract not amenable to curative surgical treatment strategies according to an Interdisciplinary Board of oncologists, surgeons, radiologists, and radiooncologists.

Other criteria for inclusion were at least one bidimensionally measurable lesion (ascites and pleural effusions were not considered measurable), Eastern Cooperative Oncology Group (ECOG) performance status (PS) ⩽2, age 18–75 years, adequate bone marrow (leucocyte count ⩾3.5 × 10^9^ per l, platelet count ⩾100 × 10^9^ per l), renal (creatinine-clearance ⩾60 ml min^−1^) and hepatic function (total serum bilirubin <2.0 mg per 100 ml, transaminases <2 × the upper limit of normal), and no prior cytotoxic treatment.

The trial protocol was approved by the local ethics review boards of all participating Hospitals, and written consent was obtained from all patients before any study-specific procedures.

### Patient evaluation

Before entry onto study, all patients underwent a full medical history, physical examination, PS, and laboratory evaluation. In addition, baseline evaluation included electrocardiograms, chest X-rays, abdominal ultrasounds, and computed tomography scans (alternatively magnetic resonance imaging scans) of the abdomen. Further imaging investigations were added if clinically indicated or for disease assessment.

Objective tumour evaluation for response was performed according to World Health Organization (WHO) standard criteria (World Health Organization, 1979). A complete response (CR) was defined as disappearance of all measurable or evaluable disease with the absence of any new lesions for at least 4 weeks. A PR was defined as a >50% reduction in the sum of the products of the largest perpendicular diameters of all measurable lesions with the absence of any new lesions for at least 4 weeks. Stable disease was defined as a reduction of <50% or an increase of <25% of measurable lesions according to the previous method, with the absence of any new lesions. Progressive disease (PD) was characterised by an increase of >25% of measurable lesions according to the previous method or the appearance of new lesions. A baseline radiographic tumour evaluation was performed within 2 weeks before treatment started and repeated after two cycles, and again after every further three cycles by the same imaging method that was used for baseline measurement. Additional evaluations were performed if PD was suspected.

Clinical response assessment was determined separately for patients with either GBC/ECC or ICC, because these two distinct presentations have been supposed to differ substantially from each other according to clinical behaviour (hilar CCCs (Klatskin tumours) have been categorised as ECC). Accordingly, patients were entered prospectively into two nonrandomised parallel groups based on location of the primary (GBC/ECC *versus* ICC) in order to evaluate the outcome more accurately. Moreover, subgroup analysis of hilar *versus* distal CCCs was performed.

Toxicity was assessed using the National Cancer Institute Common Toxicity Criteria (NCI-CTC), except for neurologic toxicity, that was graded according to Lévis scale.

### Treatment schedule

Patients received, on outpatient basis, the CAPOX regimen as reported previously (André *et al*, 1999). In brief, capecitabine 1000 mg per m^2^ per dose was administered orally twice a day on days 1–14, followed by a 7-day treatment-free period. Oxaliplatin, 130 mg m^−2^, was administered as a 2-h infusion on day 1, in a 3-week cycle. All patients received, as concomitant antiemetic prophylaxis, a 5-HT 3 antagonist intravenously prior to each oxaliplatin dose.

Treatment was terminated in the case of disease progression, intolerable side effects, or patient's choice to discontinue the therapy.

To prevent oxaliplatin-induced neurotoxicity, all patients received carbamazepine (initially 100 mg b.i.d. to achieve a serum-level of 3–6 mg l^−1^) according to the preliminary results of a pilot study (Lersch *et al*, 2002).

### Toxicity and dosage modification guidelines

The toxicities were graded according to NCI-CTC (version 2). Capecitabine doses should be interrupted in cases of grade 2 or higher events (except alopecia) and treatment should be delayed until complete recovery or until the adverse event improved to grade 0 or 1. Capecitabine was decreased by 25% in subsequent cycles at the first occurrence of a grade 2 or 3 toxicity, and it was reduced by 50% at the second occurrence of a given grade 2 or grade 3 toxicity or at the first occurrence of a grade 4 event. Treatment with capecitabine was permanently stopped if, despite dose reduction, a given toxicity occurred for the third time at grade 2 or grade 3, or a second time at grade 4.

Oxaliplatin was reduced by 25% in subsequent cycles for grade 3 or 4 neutropenia or thrombocytopenia, grade 3 diarrhoea or mucositis, any other drug-related grade 3 toxicity or painful paresthesias lasting 8–14 days (without functional impairment) (grade 2 according to Lévis scale), whereas oxaliplatin was diminished by 50% if persistent (>14 days) paresthesias (without functional impairment) emerged (grade 3). Oxaliplatin was stopped if a beginning functional impairment occurred (grade 4).

If more than two dose reductions were indicated and/or treatment was delayed for more than 3 weeks, patients were removed from the study.

### Statistical considerations

The main objective of the study was the response rate (CR and PR) according to WHO criteria. Both cohorts (groups A and B) were analysed separately according to Simon's two-stage design (Simon, 1989). If two or less CR or PR were noted in the first 18 patients of a group, accrual would stop in this cohort, otherwise it was planned to accrue at least 43 patients in this group to determine outcome more accurately. Assuming that about 5% of patients could not be evaluable, the accrual was planned to continue to a total of 47 patients for the second step of a group. The significance level was 5% and the power was 80%.

Secondary objectives were safety, OS, and time to progression (TTP). Overall survival or TTP were calculated both from the initiation of chemotherapy until death and from the date of objectively measured disease progression till the occurrence of death from any cause. The cutoff was 30 April 2007. Statistical analysis was performed using SPSS for Windows 11.5 (SPSS Inc., Chicago, IL, USA). Median OS and TTP were estimated according to the Kaplan–Meier method (Kaplan and Maier, 1959).

## RESULTS

### Patient characteristics

A total of 66 patients (27 men and 39 women) were enrolled onto this multicenter trial at the University Hospitals of Berlin, Frankfurt, Mannheim and Tübingen, Germany, and 65 patients were evaluable for efficacy and safety. One patient was retrospectively found to have a diagnosis of pancreatic adenocarcinoma and has been disclosed from the study. Patient characteristics are detailed in Table 1.

The median age of the study population was 61 years (range, 28–74). Twenty-seven, 20, and 18 had adenocarcinoma of the gallbladder, ECC, or ICC, respectively.

In group A (GBC or ECC), three patients (6%) had locoregional advanced disease. Forty-four patients (94%) had metastatic disease. Sixteen patients had one metastatic site (34%), and 28 patients (60%) had two affected sites and more. Liver involvement was noted in 39 patients (83%). In group B (ICC), four patients (22%) had one metastatic site, and 14 patients (78%) had two affected sites and more (Table 1).

In groups A and B, 21 and 5 patients, respectively, experienced disease recurrence or metastatic spread to distant organs after they had undergone curatively intended surgery previously.

### Efficacy

In group A, 2 of the 47 patients (4%) achieved a complete remission, 11 patients (23%) experienced a partial remission, and SD was noted in 23 patients (49%). Accordingly, the overall disease control rate (CR, PR, or SD) on 47 patients with GBC or ECC was 77%, and PD was found in 11 patients (23%). From initiation of chemotherapy, actuarial estimation of the median TTP and median OS were 6.5 months (95% CI, 5.3–7.7 months), and 12.8 months (95% CI, 10.0–15.6 months), respectively. At the cutoff of the study, two patients of group A were still alive.

In group B, no CR or PR was observed in 18 patients with ICC, but six patients (33%) had SD, and 12 patients (68%) experienced PD. Median TTP and OS were 2.2 months (95% CI, 1.4–3.0 months) and 5.2 months (95% CI, 0.6–9.8 months), respectively. At the cutoff of the study, all patients of group B have died.

### Subgroup analysis for efficacy

The results of the response rates for patients with GBC *versus* ECC are detailed in Table 2.

**Figure 1:**
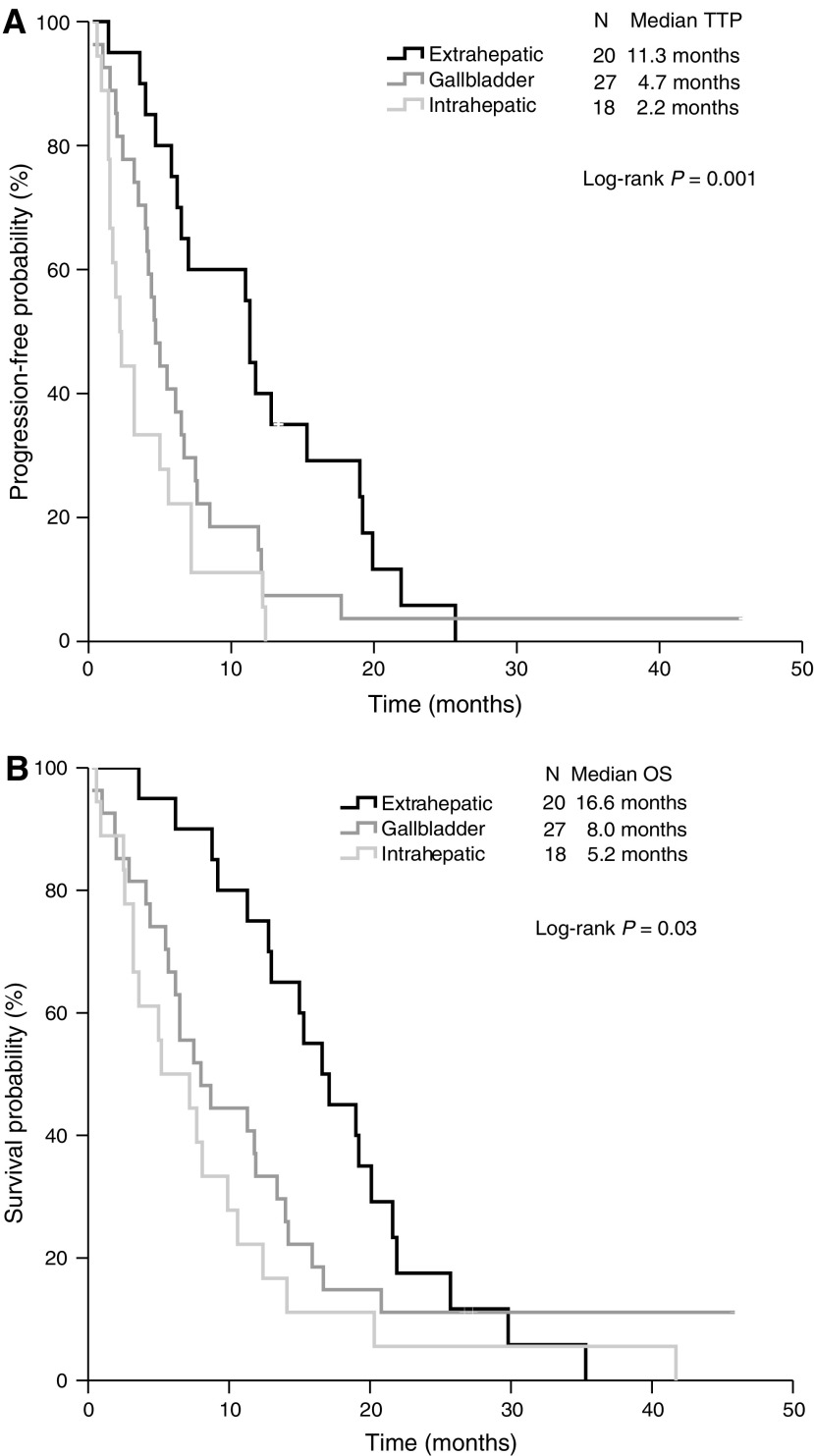
The Kaplan–Meier curves of (**A**) TTP and (**B**) OS.

Furthermore, survival analysis for patients with GBC *versus* ECC revealed markedly differences on both TTP (Figure 1A; Kaplan–Meier curves for TTP) and OS (Figure 1B; Kaplan–Meier curves for OS). Thus, TTP or OS in GBC and ECC were 4.7 months (95% CI, 3.7–5.7) and 11.3 months (95% CI, 10.6–12.0), respectively, or 8.0 months (95% CI, 4.3–11.7) and 16.6 months (95% CI, 12.7–20.5), respectively. Subgroup analysis of patients with hilar *versus* distal CCC showed no significant differences on TTP or OS. Thus, TTP or OS in hilar and distal CCC were 6.5 months (95% CI, 5.1–7.9) and 11.3 months (95% CI, 10.4–12.2), respectively, or 20.1 months (95% CI, 12.2–28) and 15.3 months (95% CI, 8.7–21.9), respectively (log-rank test, *P*=0.85, or *P*=0.51).

Subgroup analysis of patients from groups A and B revealed that TTP or OS was worst for patients with ICC compared to patients with ECC or GBC (log-rank test; *P*=0.01, or *P*=0.03, respectively) (Figure 1A and B).

### Safety

All 65 patients were evaluable for toxic effects. A total of 367 courses of treatment have been administered (median, five courses per patient; range, 1–17). The median cumulative doses for oxaliplatin and capecitabine were 560 mg m^−2^ (range, 130–1.945) and 8.500 mg m^−2^ (range, 2.000–30.500) corresponding to a relative dose intensity of 86% for oxaliplatin and 85% for capecitabine, respectively. In 46.2% of the patients, at least one cycle of treatment was delayed due to oxaliplatin and/or capecitabine-related side effects resulting in dose reductions for oxaliplatin and capecitabin in 30.8 and 46.2% of the patients, respectively. The recorded toxicities are summarised in Table 3.

The National Cancer Institute Common Toxicity Criteria grade 4 thrombocytopenia or febrile leukopenia was observed in one patient each (2% of patients, respectively). Two patients (3%) experienced grade 4 infection and a thrombembolic event grade 4 was reported in a single patient (2%). Grade 3 adverse events were thrombocytopenia (9% of patients), nausea/vomiting (6%), diarrhoea (5%), and hand–foot syndrome (5%). We observed no line infections. The most common toxicity was peripheral neuropathy observed in 54 of the 65 patients (83%). However, grade 3 or 4 peripheral sensory neuropathy was encountered only in 11 of the patients (17%) and 11% of courses (Table 4).

In two patients, study medication has been stopped due to oxaliplatin-related allergic reactions occurring in cycles 1 and 6. Generalised rash and fever have been recovered completely.

In one patient with ICC, chemotherapy was stopped after the first treatment cycle due to progression-related cholangiosepsis not amenable to stenting procedures. Despite treatment with antibiotics, he died from septic shock.

Another patient with a history of long-standing insulin-dependent diabetes mellitus died due to a cerebral ischaemic insult after the first treatment cycle. This event has been considered unlikely related to study treatment, but a causal relation with the study medication cannot be excluded entirely.

### Second-line treatment

Thirty-three patients (51%) underwent second-line chemotherapy after treatment with the CapOx regimen failed (31, gemcitabine monotherapy; 1, gemcitabine plus capecitabine; 1, mitomycin C plus capecitabine). None of the patients achieved a PR or a CR after second-line therapy.

## DISCUSSION

Although there is no standard of care in advanced biliary system adenocarcinoma, palliative chemotherapy to date remains the primary therapeutic approach. Nevertheless, the search continues for an appropriate cytotoxic treatment protocol for biliary tract carcinoma. Moreover, it remains unclear if patients with GBC or ECC may respond similarly to cytotoxic treatment as those with ICC.

In the present prospective phase II trial, stratified prospectively into two groups based on location of the primary (GBC or ECC *versus* ICC), a response rate of 27% was achieved in 47 patients with GBC or ECC (group A) (including 4% complete remissions) adding to a disease-control rate (responses and SD) of 77%. Moreover, response rates were comparable for patients with ECC and GBC (30 and 25%).

In patients with ICC, SD was found in 33% of 18 cases, whereas no objective tumour response could be detected in this subgroup.

Our data show that the CAPOX regimen may be active, particularly in the subset of ECC and GBC, although it has only modest activity in ICC.

This agrees with data from a recent trial using gemcitabine combined with platinum-based cytotoxic treatment in 42 patients with biliary system adenocarcinomas, which also demonstrated both comparable response rates for patients with ECC and GBC and a marked difference in response rates between GBC and ECC *versus* ICC (40 and 43 *versus* 7%, respectively) (Table 5) (Harder *et al*, 2006). The relatively higher response rates in the latter study as compared to our data may reflect the use of a different evaluation system (RECIST *versus* WHO criteria). In addition, accumulating data suggest that GBC in comparison to ICC responds noticeably better to various cytotoxic protocols (Table 5) (Kim *et al*, 2003; André *et al*, 2004; Patt *et al*, 2004; Alberts *et al*, 2005). These differences may reflect site-related distinct biologic features (Jarnagin *et al*, 2006).

Of note, only in studies evaluating ICC and ECC as one entity, classified as CCC, no obvious differences in response rates have been found between CCC and GBC. It may be hypothesised that the majority of responders in these studies were patients with ECC (Table 5) (Knox *et al*, 2005; Verderame *et al*, 2006). In this context, it should be emphasised that Klatskin tumours are misclassified as ICC in a substantial number of cases (Welzel *et al*, 2006).

In our series, it was most striking that median OS for patients with ECC was about twice as long than for patients with GBC (16.8 *versus* 8.1 months). Although the comparison of results of non-randomised phase II trials regarding survival may be inconclusive, that is, due to patient selection bias, our observation of an OS of 8.1 months for GBC was in good accordance with other phase II trials using fluoropyrimidin-based therapies, platin-analogues, or gemcitabine-based schedules (Table 5). A possible explanation for a shorter survival for GBC compared to ECC may be the more aggressive natural course of GBC, and that patients with ECC might also benefit from local treatment (i.e., stenting procedures). Conversely, one study found very long median survival times for GBC (16 months) using a combination chemotherapy of oxaliplatin plus gemcitabine (André *et al*, 2004). However, two other studies using this combination protocol could not reproduce this result, possibly due to differences in patient selection, inclusion criteria, and/or applications of the drugs (Harder *et al*, 2006; Verderame *et al*, 2006).

Therefore, randomised trials are warranted in biliary system adenocarcinomas, stratified according to the three entities, GBC, ICC, and ECC, to draw definitive conclusions about differences in response rates and survival.

Fifty-one percent of our patients received second-line treatment, but no complete or partial responses were seen in this setting. Accordingly, an influence of second-line treatment on outcome seems to be inferior.

The toxicity profile of the CAPOX regimen was generally favourable. Gastrointestinal grade 3/4 toxicities were nausea/vomiting and diarrhoea, each of which affected four patients (6%) including grade 4 diarrhoea in only one patient (2%) (Table 3), comparing favourably to other trials using the CAPOX regimen for treatment of gastrointestinal tumours other than biliary (Park *et al*, 2006). The most common grade 3/4 haematological event was thrombocytopenia (11% of patients), including a grade 4 event in a single patient (2%). No bleeding disorders have been seen.

In the current study, we observed grade 2 infection in 10 patients and grade 4 infection in 2 patients, respectively, causing treatment delays in only 4% of the courses. In contrast, Harder *et al* (2006) found treatment delays in 9% of cycles using a regimen with gemcitabine combined with oxaliplatin. Of note, in four of our patients, infection was based on occlusion of biliary stents, and in eight patients, no such association was found. Furthermore, in one patient with and in one patient without stent insertion, cholangiosepsis occurred.

However, no line infections were observed. In contrast, in a previous study on advanced biliary cancers using a schedule based on protracted infusion of 5-FU, rates of central line infection and venous thrombosis were 19 and 7% (Knox *et al*, 2004) indicating the risks of implantable devices.

Two patients who were withdrawn from our study due to an allergic reaction to oxaliplatin recovered fully. This oxaliplatin-related toxicity, typically occurring after application of 7–12 treatment cycles, was estimated to affect about 2% of patients (Tournigand *et al*, 1998). In addition, three patients (5%) developed grade 3 hand–foot syndrome.

The main grade 3/4 toxic effect was peripheral sensory neuropathy, which was noted in 11 patients (17%) (Table 4). However, this oxaliplatin-related side effect was reported to be completely reversible after omission of oxaliplatin in more than 80% of the patients within 8 months (Gamelin *et al*, 2002).

In conclusion, the CAPOX regimen has a favourable safety profile and is more convenient than infusional regimens, avoiding the need for indwelling devices and frequent hospital visits. Finally, the prospective data presented here may open a triad perspective on adenocarcinomas of the biliary system after platinum-based cytotoxic treatment. First, response rates and outcome seem worst for patients with ICC compared to patients with ECC or GBC, indicating that tumour growth in these neoplasms may reflect other biological mechanisms than in GBC and/or ECC; Second, GBC may have an intermediate prognosis with response rates very similar to ECC, whereas outcome might be poorer. Third, ECC, including perihilar CCC, may have high response rates and median survival times that clearly tend to exceed those of GBC and ICC. To prove these hypotheses, prospective randomised trials should be undertaken.

## Figures and Tables

**Table 1:** Patient characteristics by treatment group and site of the primary

	**Group A**		**Group B**
**Characteristic**	**Gallbladder**	**Extrahepatic bile duct**	**Intrahepatic bile duct (mass-forming-type)**
No. of patients	27	20	18
*Age (years)*			
Median	61	66	52
Range	49–73	49–74	28–74
			
*Sex* (%)			
Male	9 (33)	10 (50)	8 (44)
Female	18 (67)	10 (50)	10 (56)
			
*PS, ECOG* (%)			
0	8 (30)	7 (35)	7 (39)
1	15 (55)	11 (55)	9 (50)
2	4 (15)	2 (10)	2 (11)
			
*Sites of disease* (%)			
Locoregional	0 (0)	3 (15)	0 (0)
Liver	25 (93)	14 (70)	18 (100)
Lymph node	14 (52)	5 (25)	12 (67)
Lung	2 (7)	2 (10)	6 (33)
Peritoneum	1 (4)	1 (5)	0 (0)
Other	6 (22)	7 (35)	2 (11)
			
*No. of metastatic sites* (%)			
1	10 (37)	6 (30)	4 (22)
⩾2	17 (63)	11 (70)	14 (78)
			
*Prior surgery* (%)			
Yes	12 (44)	9 (45)	5 (28)
No	15 (56)	11 (55)	13 (72)
			
*CEA elevated* (%)			
Yes	6 (22)	6 (30)	7 (39)
No	21 (78)	13 (65)	9 (50)
Not done	0 (0)	1 (5)	2 (11)
			
*CA 19-9 elevated* (%)			
Yes	19 (70)	16 (80)	10 (56)
No	8 (30)	4 (20)	6 (33)
Not done	0 (0)	0 (0)	2 (11)

**Table 2:** Best response to treatment

		**Group A**			
		**Extrahepatic bile duct carcinoma (*n*=20)**			
**Outcome**	**Gallbladder carcinoma (*n*=27)**	**Hilar (*n*=6)**	**Distal (*n*=14)** **No. of patients (%)**	**Hilar and distal (*n*=20)**	**Group B** **Intrahepatic bile duct carcinoma (*n*=18)**
CR	1 (4)	0 (0)	1 (7)	1 (5)	—
PR	7 (26)	2 (33)	2 (14)	4 (20)	—
SD	9 (33)	3 (50)	11 (79)	14 (70)	6 (33)
PD	10 (37)	1 (17)	0 (0)	1 (5)	12 (67)
Disease control rate (CR or PR or SD)	36 (77)				6 (33)

Abbreviations: CR=complete remission; PD=progressive disease; PR=partial remission; SD=stable disease.

**Table 3:** Toxicity in any cycle (worst per patient) (both patient series (groups A and B), *n*=65)

	**Grade 2**		**Grade 3**		**Grade 4**	
	**No. of patients**	**%**	**No. of patients**	**%**	**No. of patients**	**%**
Neutropenia	9	14	0	0	1	2
Febrile neutropenia	0	0	0	0	1	2
Thrombocytopenia	13	20	6	9	1	2
Anemia	12	18	0	0	0	0
Nausea/vomiting	19	29	4	6	0	0
Diarrhoea	12	18	3	5	1	2
Mucositis	1	2	0	0	0	0
Hand–foot syndrome	6	9	3	5	NA	NA
Infection	10	15	0	0	2	3
Thrombembolic events	0	0	0	0	1	2

Abbreviation: NA=not applicable.

**Table 4:** Incidence of neurosensory symptoms according to Lévís scale^a^ (worst per patient) (both patient series, group A and B), *n*=65

**Paresthesia** a	**Grade 1**		**Grade 2**		**Grade 3**		**Grade 4**	
No of patients (%)	20	31	23	35	10	15	1	2
No of cycles (%)	53	14	48	13	39	11	1	0.3

a Lévís scale: grade 1, paresthesias of moderate intensity lasting less than 7 days; grade 2, painful paresthesias lasting 8–14 days (without functional impairment); grade 3, persistent (>14 days) paresthesias (without functional impairment); grade 4, beginning functional impairment.

**Table 5:** Summary of phase II trials of biliary system adenocarcinoma (trials selected differ between GBC, ECC, and/or ICC)

			**No. of patients RR (%)**				**Median survival (months)**		
**Author (year)**	**Regimen**	**Metastatic disease %**	**Total**	**GBC**	**ECC**	**ICC**	**GBC**	**ECC**	**ICC**
André *et al* (2004)	Gemcitabine Oxaliplatin	94	31^†^	11 (54)	4 (25)	16 (21)	16	NA	14.5
Kim *et al* (2003)	Capecitabine Cisplatin	67	38^*^	19 (32)	9 (11)	14 (14)		9.1	
Patt *et al* (2004)	Capecitabine	NA	26^†^	8 (50)	18 (6)		9.9	8.1	
Knox *et al* (2005)	Gemcitabine Capecitabine	89	42^*^	22 (28)	23 (34)		6.6	19	
Alberts *et al* (2005)	Gemcitabine 5-FU/LV	83	42^*^	14 (21)	9 (7)	19	7.2	9.9	
Verderame *et al* (2006)	Gemcitabine Oxaliplatin	67	24^*^	9 (44)	15 (53)	6.0		12.0	
Harder *et al* (2006)	Gemcitabine Oxaliplatin	90	31^*^	10 (40)	7 (43)	14 (7)	NA	13.3	8.4
This study	Capecitabine Oxaliplatin	95	65^†^	27 (30)	20 (25)	18 (0)	8.2	16.8	5.2

Abbreviations: ECC=extrahepatic cholangiocarcinoma; GBC=gallbladder carcinoma; ICC=intrahepatic mass-forming type cholangiocarcinoma; NA=data not available; RR=response rate; ^†^Response evaluation according to WHO (World Health Organization) criteria; ^*^Response evaluation according to RECIST (Response evaluation in Solid Tumors) criteria.
